# Functional changes in the auditory cortex and associated regions caused by different acoustic stimuli in patients with presbycusis and tinnitus

**DOI:** 10.3389/fnins.2022.921873

**Published:** 2022-10-19

**Authors:** Jakub Fuksa, Oliver Profant, Jaroslav Tintěra, Veronika Svobodová, Diana Tóthová, Antonin Škoch, Josef Syka

**Affiliations:** ^1^Department of Otorhinolaryngology, 3rd Faculty of Medicine, Faculty Hospital Královské Vinohrady, Charles University, Prague, Czechia; ^2^Department of Auditory Neuroscience, Institute of Experimental Medicine, The Czech Academy of Sciences, Prague, Czechia; ^3^MR Unit, Institute for Clinical and Experimental Medicine, Prague, Czechia; ^4^Department of Otorhinolaryngology, Head and Neck Surgery, 1st Faculty of Medicine, Charles University in Prague and University Hospital Motol, Prague, Czechia

**Keywords:** presbycusis, tinnitus, auditory system, limbic system, functional MRI

## Abstract

Presbycusis and tinnitus are the two most common hearing related pathologies. Although both of these conditions presumably originate in the inner ear, there are several reports concerning their central components. Interestingly, the onset of presbycusis coincides with the highest occurrence of tinnitus. The aim of this study was to identify age, hearing loss, and tinnitus related functional changes, within the auditory system and its associated structures. Seventy-eight participants were selected for the study based on their age, hearing, and tinnitus, and they were divided into six groups: young controls (Y-NH-NT), subjects with mild presbycusis (O-NH-NT) or expressed presbycusis (O-HL-NT), young subjects with tinnitus (Y-NH-T), subjects with mild presbycusis and tinnitus (O-NH-T), and subjects with expressed presbycusis and tinnitus (O-HL-T). An MRI functional study was performed with a 3T MRI system, using an event related design (different types of acoustic and visual stimulations and their combinations). The amount of activation of the auditory cortices (ACs) was dependent on the complexity of the stimuli; higher complexity resulted in a larger area of the activated cortex. Auditory stimulation produced a slightly greater activation in the elderly, with a negative effect of hearing loss (lower activation). The congruent audiovisual stimulation led to an increased activity within the default mode network, whereas incongruent stimulation led to increased activation of the visual cortex. The presence of tinnitus increased activation of the AC, specifically in the aged population, with a slight prevalence in the left AC. The occurrence of tinnitus was accompanied by increased activity within the insula and hippocampus bilaterally. Overall, we can conclude that expressed presbycusis leads to a lower activation of the AC, compared to the elderly with normal hearing; aging itself leads to increased activity in the right AC. The complexity of acoustic stimuli plays a major role in the activation of the AC, its support by visual stimulation leads to minimal changes within the AC. Tinnitus causes changes in the activity of the limbic system, as well as in the auditory AC, where it is bound to the left hemisphere.

## Highlights

- Tinnitus leads to increased involvement of the left AC.
- Tinnitus alters the activity within the structures of limbic system.
- Complexity of acoustic stimulus alters the employment of the ACs.
- Age related hearing loss leads to lower activation of the AC.

## Introduction

Age related hearing loss (presbycusis) is a progressive, bilateral, and in most cases symmetrical, sensorineural, hearing deterioration. It has multifactorial etiology and affects both the peripheral and central auditory systems (Gates and Mills, 2005). Approximately one-third of the population above 65 years of age experience presbycusis (Lin et al., 2011). The traditional view of presbycusis is a pathology of the auditory periphery. Based on the morphological characteristics, four types of presbycusis were originally described: sensory, where the main feature is the direct damage of hair cells and supporting cells; neural, with loss of spiral ganglion neurons; metabolic, characterized by an atrophy of the stria vascularis, and spiral ligament and mechanical, defined by a stiffening of the basilary membrane (Schuknecht, 1974).

Presbycusis is currently considered to be a complex manifestation of aging, resulting in hearing impairment as a combination of the hypofunction of both the peripheral and central parts of the auditory system (Working Group on Speech Understanding, 1988). Ouda et al. (2015) summarized some of the age related changes within the central auditory system in their review, including the decreased activity of the inhibitory systems and changes in the activity of calcium-binding proteins, particularly parvalbumin. Morphometric changes as a reduction of gray matter volume, decreased cortical surface area and thinning of the AC, as reported in a human MRI study (Profant et al., 2014) are further signs of central presbycusis. Among the dominant audiometric parameters affected by presbycusis is the deteriorated ability to detect the temporal features of sound (Grose and Mamo, 2010), that eventually leads to a worsening of speech understanding, especially in a noisy environment (Arons, 1992).

Tinnitus is a conscious auditory perception without the presence of corresponding external sound stimuli. The reported prevalence of tinnitus is between 10% and 15% of the adult population (Baguley et al., 2013), increasing with age, and it is slightly higher in men compared to women (Heller, 2003). For an estimated 20% of these patients, tinnitus is a heavy burden on their quality of life and a possible cause of insomnia, depression, or concentration difficulty (Smits et al., 2007; Piccirillo et al., 2020).

Subjective tinnitus and sensorineural hearing loss are closely connected, and most people with tinnitus also suffer from hearing loss. However, approximately 20–30% of patients with tinnitus have only minor or no clinically identified hearing impairment (Stouffer and Tyler, 1990) and, on the other hand, there are patients with hearing loss who do not suffer from tinnitus; epidemiology data show a bigger prevalence of hearing loss compared to the presence of self-reported tinnitus (Eggermont, 2017).

Although tinnitus and presbycusis can be caused by changes of peripheral as well as central origin, the pathophysiology behind both entities is most likely different. Cochlear injury can lead to deafferentation of the central auditory structures which results in neural changes that are believed to underlie the sensation of tinnitus. These changes comprise increased synchronous neural activity, an increased firing rate, and potentially changes in cortical tonotopic maps (Eggermont and Roberts, 2012), however, the last effect remains controversial (Langers et al., 2012; Koops et al., 2020). Alternatively, several situations cannot be induced by cochlear (i.e., peripheral) pathology only. Patients who underwent vestibular schwannoma surgery, that led to a surgical neurectomy of the cochlear nerve, described worsening of tinnitus after surgery, despite the disconnection of the inner ear from the central auditory system (House and Brackmann, 1981).

Another aspect of presbycusis and tinnitus is their negative effect on cognitive abilities due to overloading of the cognitive resources by either being a distractor (tinnitus), or by the need for cognitive compensation of a disrupted speech signal (presbycusis) as described in the load theory by Lavie et al. (2004). Therefore, the combination of tinnitus and presbycusis provides an intriguing option to identify such an effect on the function of the AC, and whether the presence of another distractor (incongruent visual stimulus) leads to additional changes in the AC involvement.

In this study, we aim to identify changes in the brain activity evoked by different types of acoustic stimulations and the combination of acoustic and visual stimulation within the central auditory systems using a 3T fMRI system; along with the effect of aging, hearing loss (different degree of presbycusis), and the presence of tinnitus. Consequently, young and elderly patients with and without tinnitus and hearing loss were enrolled in this study. As the main problem of presbycusis, influenced by cognition and tinnitus, is believed to be speech processing, we chose complex auditory stimuli with different degrees of acoustic information for auditory stimulation. Regarding the regions of interest, we focused on the AC and other structures within the central nervous system, that were previously identified as part of the tinnitus network – amygdala (Amg), hippocampus (HP) and insula (Ins) (Husain, 2016; Leaver et al., 2016; Profant et al., 2020; Khan et al., 2021). Additionally De Ridder et al. (2014) correlated the level of tinnitus distress with insular activity.

For a better overview of the expected changes at the level of the AC we chose to involve lateralization index (LI) in our analysis, mainly due to the difference in involvement of left and right ACs in the processing of complex sounds and how it changes with aging, hearing loss (Profant et al., 2015; Tremblay et al., 2021), and tinnitus. The function of the AC is lateralized; the left AC is believed to be involved in the temporal processing (speech processing), whereas the right AC is more spectrally oriented which is important for music (Schönwiesner et al., 2007) – however, such a specialization diminishes with aging (Keller et al., 2019).

## Materials and methods

Based on the inclusion criteria [presence of tinnitus (T, tinnitus; NT, no tinnitus) and age (Y, young, O, old)] four groups were formed, with the two latter divided based on the degree of presbycusis [the expressed presbycusis – hearing loss was present in the case of auditory threshold elevation more than average hearing loss according to volunteers age + 2xSD (Jilek et al., 2014)], NH – normal hearing, HL – hearing loss; the same approach was also used in our previous study (Profant et al., 2020). Overall, 78 volunteers were enrolled into the study and were divided into the following groups Y-NH-NT (15 young volunteers with normal hearing and no tinnitus, seven males and eight females, mean age 26 ± 0.7 years), Y-NH-T (15 young patients with present tinnitus and normal hearing (Y-NH-T group), 11 males and 4 females, mean age 32 ± 2.6 years.), O-NH-NT (12 patients with mild presbycusis, four males and eight females; mean age 60 ± 4.1 years), O-NH-T (12 patients with present tinnitus and mild presbycusis, four males and eight females; mean age 65 ± 1.8 years), O-HL-NT [12 elderly patients with expressed presbycusis, eight males and four females; age 72 ± 2.1 years (mean ± SEM)] and O-HL-T (12 patients with both present tinnitus and expressed presbycusis, seven males and five females; mean age 68 ± 2.1 years).

All participants declared no previous otologic surgery: vestibular lesion, chronic exposure to loud noise, severe head trauma, lesion of the facial nerve, disorder of the cervical spine, or had a self-reported central nervous system disorder. None of the participants were musical professionals, but several in the elderly group played musical instruments sporadically (not more than once a month). An otoscopic examination, with removal of the cerumen and confirmation of an intact tympanic membrane, was performed on all of the participants. The examination procedures were approved by the Ethics Committee of the University Hospital Motol, in Prague. All participants signed written informed consent.

### Audiometric and tinnitus examination

#### Pure tone audiometry

For the basic audiometric examination, pure tone audiology was used with the aim to evaluate the hearing thresholds of each volunteer, and to include them into specific groups for further analysis. The examination was carried out on our custom-made device (operated by software package built in Matlab environment) at the following frequencies 0.125, 0.25, 0.5, 0.71, 1, 1.6, 2, 3.15, 4, 6.3, 8, 10, 12.5, and 16 kHz, with a resolution of 2 dB. Acoustic stimuli were presented separately into each ear *via* Sennheiser HDA 200 high-frequency audiometric headphones. The equipment was calibrated according to ISO 389-5, ISO 389-8, ISO 8253-3, and IEC 60645-3 standards, using the Brüel and Kjaer 4153 Artificial Ear. For easier interpretation of the audiometric data, the pure tone average (PTAV, an average of hearing thresholds on both ears) from all the frequencies was calculated.

#### Speech audiometry

Speech audiometry in quiet (close set of 10 words) was tested using our custom-made device. The acoustic stimuli were presented *via* Sennheiser HDA 200 headphones, simultaneously into both ears. At each measured intensity 10 words were presented, and the speech recognition score (percentage of understood words) was registered. Speech audiometry in babble noise, according to Dlouhá et al. (2012), was measured with background noise set at 63 dB SPL and speech intensity set at 65 dB SPL. The background noise was increased by 3 dB steps (for each trial 10 words were delivered) with the aim to reach the intensity of background noise that would lead to 50% speech recognition.

#### Tinnitus characteristics

The presence (or absence) of tinnitus was self -reported by the patients. In all patients with the presence of tinnitus (thus further included in the O-HL-T, O-NH-T, and Y-NH-T group), tinnitus laterality, length, and annoyance were reported.

In each group, there was an approximately equal portion of patients who lateralize tinnitus into a given ear, and patients without tinnitus lateralization (i.e., in both ears and perception of sound “in the head”). In the Y-NH-T group, there were nine patients with non-lateralized tinnitus, five with tinnitus on the right side, one on the left side. In the O-NH-T group, six patients had non-lateralized tinnitus, three on the right and three on the left side, and in the O-HL-T group, seven patients had tinnitus non-lateralized, three on the right side and two on the left side.

The average duration of tinnitus was 3.3 ± 0.7 years in the Y-NH-T group, 3.8 ± 0.7 in the O-NH-T group, and 5.3 ± 0.9 years in the O-HL-T group. The differences in the tinnitus durations were not statistically significant (one-way ANOVA; *p* = 0.166).

The mean tinnitus annoyance was established by the Tinnitus Handicap Inventory (THI) (Newman et al., 1996), and the degree of discomfort caused by the presence of tinnitus was classified into five categories (McCombe et al., 2001) slight (S)− participants, mild (Mi)− participants, moderate (Mo)− participants, severe (Sev)− participant, catastrophic (C)−0 participants. In Y-NH-T, eight patients had slight (S), five mild (Mi), one moderate (Mo), and one severe (Sev), and zero participants with catastrophic (C). In O-NH-T, nine patients had (S), two (Mi), and one (Mo) tinnitus. No patient had (Sev) or (C) tinnitus. In the O-HL-T group, eight patients had (S), three (Mi), zero (Mo), and one patient (Sev). No patient had (C).

### Cognitive impairment testing

All of the elderly patients were tested by Montreal Cognitive Assessment (MoCA) test. The average score was 27.5 ± 0.6 in O-HL-NT; 26.8 ± 0.09 in O-NH-NT, 26.9 ± 0.6 in O-HL-T and 27.5 ± 0.4 in the O-NH-T group. The difference in MoCA results between the groups was non-significant (one-way ANOVA, *p* = 0.77). There were two patients in the O-HL-NT group, three patients in the O-NH-NT, two patients in the O-HL-T, and one patient in the O-NH-T group who scored less than 26, which is the threshold of mild cognitive impairment. The lowest MoCA score was 22 in the O-NH-NT group, therefore no participant suffered from clinically relevant dementia.

### Magnetic resonance imaging examination

Functional MRI examinations were performed on a Siemens Trio 3T scanner (Siemens, Erlangen, Germany) using a 12-channel head coil and a GRE-EPI sequence: TE = 30 ms, TR = 5 s, flip angle = 90°, voxel size of 3 × 3 × 3 mm^3^, 33 slices (thickness of 3 mm). An event-related design with sparse data acquisition was used, images were acquired during the first 2 s of TR, and then the acoustic stimulation was performed (without scanner noise). For auditory stimulation, four different stimuli were used: word (closed set of words used for Czech speech audiometry), babble, un-word (pink noise modulated by amplitude envelope of word), and a combination of word + babble. Stimulation intensities for word, babble, and un-word, oscillated around 78 dB SPL, for word + babble it was 81 dB. The acoustic stimulation was delivered simultaneously into both ears by MRI compatible Confon headphones (MR Confon GmbH, Magdeburg, Germany). The visual stimulation was presented *via* data-projector into the space of magnet bore, where subjects saw projected pictures in a mirror fixed on the head coil.

Three fMRI measurements with different kinds of stimulation were performed:

(1) Sound in the form of single words with babble noise (words + babble) was delivered simultaneously with the presentation of either a congruent or incongruent picture, and the subject was instructed to recognize whether or not the picture shows the meaning of the words heard. This measurement contained 96 volumes (dynamical acquisition) during which blocks of four volumes without any stimuli, and four volumes with described stimuli, was repeated 12 times. Each block with stimuli contained both congruent and incongruent stimuli in pseudo random order, the number of both types was the same. The acquisition time was 8:07 min.
(2) The same measurement as in (1) but the stimuli were just pure words without any additional noise.
(3) The measurement combined all four types of acoustic stimulation (word, word + babble, un-word, babble) but without any visual context. Babble noise is produced by six talkers. In this case, blocks of two stimuli of the same type were used in alternation with four volumes of the remainder. Each kind of acoustic stimulation was delivered six times (the remainder of four volumes at the beginning was followed by 144 volumes, total acquisition time was 12:27 min).

### The evaluation of fMRI data

Functional MRI data was evaluated in SPM 12. Standard preprocessing contained realignment, slice timing, normalization to standard space, and spatial smoothing (Gauss kernel with FWHM of 4 × 4 × 4 mm). Statistical evaluation was performed using a general linear model, and the statistical threshold used on the subject individual level was *p* = 0.05 with FWE correction.

**FIGURE 1:**
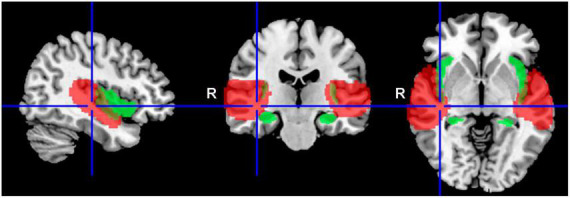
Delineation of the applied masks. AC in red (structures: part of the superior temporal gyrus, part of the transverse temporal gyrus, central and parietal operculum, planum polare, planum temporale), and tinnitus related structures in green (Amg, HP, Ins).

Various group statistics were used to compare either the differences between groups of subjects (two-sample *t*-test), or the differences between different types of stimuli (paired *t*-test). Differential statistical maps obtained with these second level statistics were masked with preselected anatomical regions, and the final uncorrected statistical threshold of *p* = 0.05 was then used. Preselected regions of interest were: auditory cortex (the mask of these functional regions was created using group statistics from all subjects, using one-sample *t*-test with *p* = 0.001 FWE threshold; this includes following the anatomical regions: part of superior temporal gyrus, part of transverse temporal gyrus, central and parietal operculum, planum polare, planum temporale) and amygdala, hippocampus and insula (this mask was selected from SPM 12 anatomical atlas, Wellcome Trust Centre for Neuroimaging, London, UK). These two masks are shown in Figure 1, auditory regions in red color, and regions including amygdala, hippocampus and insula in green color.

In addition, to asses laterality of the acoustic effect, the lateralization index (LI) was calculated with the LI toolbox for SPM. This toolbox uses the bootstrap method (Wilke and Schmithorst, 2006; Wilke and Lidzba, 2007), and finally the weighted bootstrapped LI is calculated. The default parameters of the SPM LI toolbox were used for LI calculations: sub sample size *k* = 25%, minimum sample size 5, maximum sample size 10,000, and only voxels from the temporal lobes were used.

## Results

### Auditory examination

**FIGURE 2:**
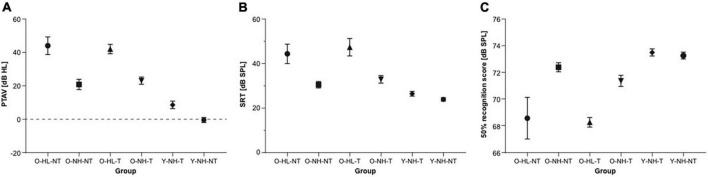
The results of the audiometric examination of all groups. **(A)** The comparison of the pure tone average among individual groups (ANOVA). **(B)** The comparison of speech reception thresholds among individual groups. **(C)** The comparison of speech in noise perception among individual groups expressed as stimulus intensity in 50% speech recognition.

The pure tone average (PTAV) was assessed as an average of the hearing thresholds in both ears. PTAV in the O-HL-NT group was 44.0 ± 5.3 dB HL, in O-NH-NT group 20.9 ± 3.0 dB HL, in O-HL-T group 42.03 ± 2.8 dB HL, in O-NH-T group 23.1 ± 2.1 dB HL, in Y-NH-T group 8.6 ± 2.3 dB HL and in Y-NH-NT group −0.2 ± 1.5 dB HL. The comparison of PTAV between all six groups shows significant differences (one-way ANOVA, *p* < 0.0001, Tukey’s multiple comparison test shows *p* < 0.0001 significant differences between O-HL-NT vs. O-NH-NT, O-HL-NT vs. Y-NH-T, O-HL-NT vs. Y-NH-NT, O-NH-NT vs. O-HL-T, O-NH-NT vs. Y-NH-NT, O-HL-T vs. Y-NH-T, O-HL-T vs. Y-NH-NT, O-NH-T vs. Y-NH-NT; *p* < 0.001 between O-HL-NT vs. O-NH-T, O-HL-T vs. O-NH-T and *p* < 0.05 between O-NH-NT vs. Y-NH-T and O-NH-T vs. Y-NH-T groups) (Figure 2A).

Two standard tests were used to assess speech perception. In speech audiometry in silence, the speech recognition threshold (SRT) is a level at which the individual understands 50% of the speech material. In the O-HL-NT group, SRT was 44.4 ± 4.4 dB SPL, in the O-NH-NT group 30.5 ± 1.5 dB SPL, in the O-HL-T group 47.4 ± 3.9 dB SPL, in the O-NH-T 33.0 ± 1.7 dB SPL, in the Y-NH-T group 26.5 ± 1.1 dB SPL, and in the Y-NH-NT group 23.9 ± 0.7 dB SPL. The comparison of SRT between all six groups shows significant differences (one-way ANOVA, *p* < 0.0001; Turkey’s multiple comparison test shows *p* < 0.0001 significant differences between O-HL-NT vs. Y-NH-T, O-HL-NT vs. Y-NH-NT, O-HL-T vs. Y-NH-T, O-HL-T vs. Y-NH-NT groups; *p* < 0.001 between O-NH-NT vs. O-HL-T group and *p* < 0.05 between O-HL-NT vs. O-NH-NT, O-HL-NT vs. O-NH-T and O-HL-T vs. O-NH-T groups) (Figure 2B).

For the speech in babble noise (SIN), the results were obtained as a level of background noise resulting in a 50% recognition score. This level was 68.6 ± 1.6 dB SPL in the O-HL-NT group, 72.4 ± 0.3 dB SPL in the O-NH-NT group, 68.3 ± 0.4 dB SPL in the O-HL-T group, 71.2 ± 0.4 dB SPL in the O-NH-T group, 73.5 ± 0.3 dB SPL in the Y-NH-T group, and 73.2 ± 0.3 dB SPL in the Y-NH-NT group. The comparison of SIN background noise level between all six groups shows significant differences (one-way ANOVA, *p* < 0.000; Tukey’s multiple comparison test, shows *p* < 0.0001 significant differences between O-HL-NT vs. Y-NH-T and O-HL-NT vs. Y-NH-NT groups; *p* < 0.05 between O-HL-NT vs. O-NH-NT, O-NH-NT vs. O-HL-T and O-HL-T vs. O-NH-T groups) (Figure 2C). All the results are listed as the mean ± SEM.

### The effect of different types of acoustic stimulation

The aim of using different types of acoustic stimulation was to identify changes in the activation of ACs in both hemispheres (part of superior temporal gyrus, part of transverse temporal gyrus, central and parietal operculum, planum polare, planum temporale) recorded by fMRI based on the different complexity (clarity) of the stimulus (rated from the most complex toward the least: word + babble, babble, un-word, word).

Significant activation of the left and right ACs was present in all the subjects, regardless of the type of acoustic stimulus (*p* = 0.05 FWE).

To better understand the involvement of the left and right AC and how it is affected by different types of acoustic stimulation, the LI was calculated as described under section “Materials and Methods.” When data from all the volunteers and all acoustic stimuli were pooled together the LI was-0.028, suggesting a slight dominance of the right AC in the processing of complex acoustic stimulation. However, the LI changed based on the type of stimulus from positive values (left side) toward negative, regarding the clarity of the stimulus (word + babble LI = 0.093, babble LI = 0.041, un-word LI = –0.028, word LI = –0.1). An interesting outcome was presented when data from the volunteers without any pathology (Y-NH-NT) were analyzed for word stimulation; the activated region was localized in the left AC (*p* = 0.05, FWE) with LI slightly shifted toward the left AC (LI = 0.044).

### The effect of aging

**FIGURE 3:**
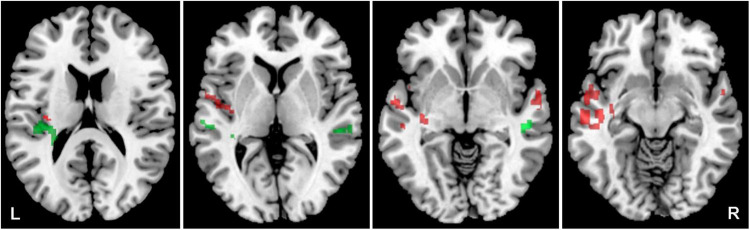
Cortical activation evoked by the combination of all acoustic stimuli within the mask of AC, the comparison of all elderly (O-NH-NZT + O-NH-T + O-HL-NT + O-HL-T) vs. young groups (Y-NH-NT + Y-NH-T). The comparison of elderly > young in red (structures: left middle temporal gyrus, central operculum), young > elderly in green (structures: left transverse temporal gyrus, left planum temporale, left superior temporal gyrus and right middle temporal gyrus), *p* = 0.05, uncorrected.

The aim was to identify the effect of aging on the activity in both ACs evoked by acoustic stimulation (all four types of stimuli combined) and recorded by fMRI. Aging leads to increased activation within the left AC, specifically in the medial temporal gyrus and central operculum (O-NH-NT + O-NH-T + O-HL-NT + O-HL-T > Y-NH-NT + Y-NH-T, *p* = 0.05, uncorrected), whereas the young subjects have slightly greater activation in the left planum temporale, left transverse temporal gyrus, and right middle temporal gyrus (Y-NH-NT + Y-NH-T > O-NH-NT + O-NH-T + O-HL-NT + O-HL-T, *p* = 0.05, uncorrected) (Figure 3 and Table 1).

**TABLE 1:** The effect of aging on the evoked activity within the structures of AC mask.

	Cluster-level		Peak-level		Peak position		
	*P* uncorr	*K*_*E*_	*P* uncorr	*T*	*X*	*Y*	*Z*
**O-NH-NT + O-NH-T + O-HL-NT + O-HL-T** ** >** **Y-NH-NT + Y-NH-T**							
Superior temporal gyrus L	0.002	615	0.000	4.64	–46	–16	–10
Middle temporal gyrus R	0.099	135	0.001	3.10	56	–20	–16
**Y-NH-NT + Y-NH-T** ** >** **O-NH-NT + O-NH-T + O-HL-NT + O-HL-T**							
Superior temporal gyrus R	0.172	90	0.000	3.55	50	–26	–4
Transverse temporal gyrus L	0.022	289	0.000	3.50	–28	–36	12

The comparison of old vs. young and young vs. old shows quantitatively where older subjects activated more (upper part) or younger subjects activated more on differential statistical maps masked with “main effect” (see description in section “Materials and methods”), uncorrected threshold of *p* = 0.05 and minimum cluster size of 50 voxels (*K_E_* is a cluster size expressed in voxels) (*K_E_* is a cluster size expressed in voxels).

Aging also affected the LI of ACs, leading to less extreme values in the elderly compared to the young (O-NH-NT + O-NH-T + O-HL-NT + O-HL-T vs. Y-NH-NT + Y-NH-T: word LI 0.053 vs. –0.011, babble LI 0.053 vs. 0.045), suggesting a more even employment of both ACs by the aged population.

### The effect of tinnitus

**FIGURE 4:**
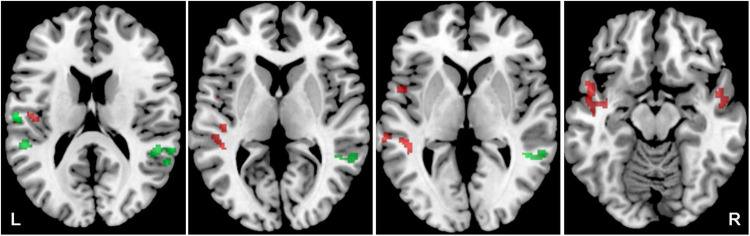
Cortical activation by a combination of all acoustical stimuli within the mask of AC, the comparison of all tinnitus (Y-NH-T + O-NH-T + O-HL-T) vs. no tinnitus (Y-NH-NT + O-NH-NT + O-HL-NT) groups. The comparison of tinnitus > no tinnitus in red (structures: left temporal gyrus, left middle temporal gyrus, left planum temporale), no tinnitus > tinnitus in green (structures: left planum temporale, left central operculum, right superior temporal gyrus, right middle temporal gyrus), *p* = 0.05 uncorrected.

**FIGURE 5:**
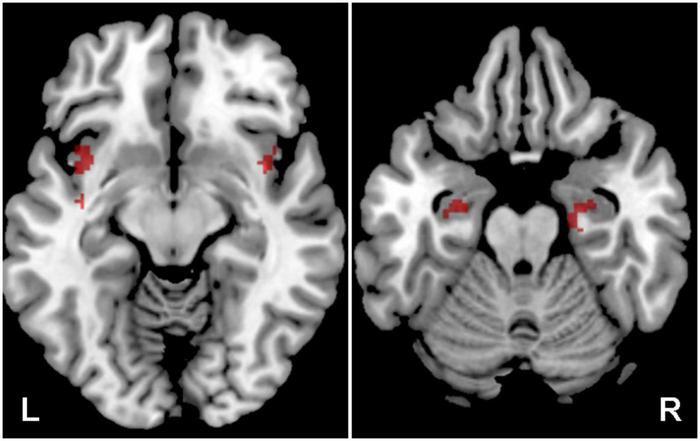
Cortical of activation by a combination of all acoustic stimuli within the tinnitus related structures (Amg, HP, Ins), the comparison of all tinnitus (Y-NH-T + O-NH-T + O-HL-T) vs. no tinnitus (Y-NH-NT + O-NH-NT + O-HL-NT) groups. The comparison of tinnitus > no tinnitus in red (structures: bilateral Ins, HP), no tinnitus > tinnitus in green (structures: no activation), *p* = 0.05 uncorrected.

The aim was to identify how the presence of tinnitus affects the activity evoked by all four acoustic stimuli. The effect of tinnitus was studied within the bilateral ACs (the LI was calculated) as well as in the tinnitus related limbic structures HP, Amg and Ins, where morphometric changes related to tinnitus presence were previously described (Profant et al., 2020). Tinnitus led to an increased activation by acoustic stimulation (all four stimuli combined) in the following regions of ACs: bilateral superior temporal gyrus (mainly left) and temporal pole, left middle temporal gyrus and planum polare (Figure 4), and bilateral Ins and HP (Figure 5) (Y-NH-T + O-NH-T + O-HL-T > Y-NH-NT + O-NH-NT + O-HL-NT, *p* = 0.05, uncorrected; Table 2), whereas the absence of tinnitus led to increased activation in the following region of ACs: the left planum temporale, central operculum and right superior and middle temporal gyrus (Y-NH-NT + O-NH-NT + O-HL-NT > Y-NH-T + O-NH-T + O-HL-T, *p* = 0.05, uncorrected; Table 2).

**TABLE 2:** The effect of tinnitus on the evoked activity within the structures of AC mask and structures of tinnitus network.

	Cluster-level		Peak-level		Peak position		
	*P* uncorr	*K*_*E*_	*P* uncorr	*T*	*X*	*Y*	*Z*
**Y-NH-T + O-NH-T + O-HL-T** ** >** **Y-NH-NT + O-NH-NT + O-HL-NT**							
Temporal pole L	0.012	367	0.000	3.69	–50	12	–22
Superior temporal gyrus L	0.087	152	0.001	3.25	–44	–38	4
Planum polare R	0.130	116	0.002	2.88	50	2	–12
Central operculum L	0.246	66	0.002	2.82	–44	–16	16
Anterior insula L	0.264	61	0.002	2.96	–30	8	–16
Hippocampus R	0.321	48	0.002	3.06	32	–10	–24
Hippocampus L	0.392	36	0.008	2.47	–26	–10	–20
Posterior insula L	0.436	30	0.001	3.12	–38	–14	–6
**Y-NH-NT + O-NH-NT + O-HL-NT** ** >** **Y-NH-T + O-NH-T + O-HL-T**							
Middle temporal gyrus R	0.029	265	0.002	2.95	58	–52	18
Central operculum L	0.272	59	0.001	3.15	–58	–16	18
Superior temporal gyrus L	0.208	78	0.001	3.12	–52	–38	14

The comparison of tinnitus vs. non-tinnitus groups shows in the upper part where patients with tinnitus activated more and where non-tinnitus subjects activated more (lower part). Differential statistical maps were masked with both described masks and uncorrected threshold of *p* = 0.05 with minimum cluster size of 30 voxels was used (*K_E_* is a cluster size expressed in voxels).

**FIGURE 6:**
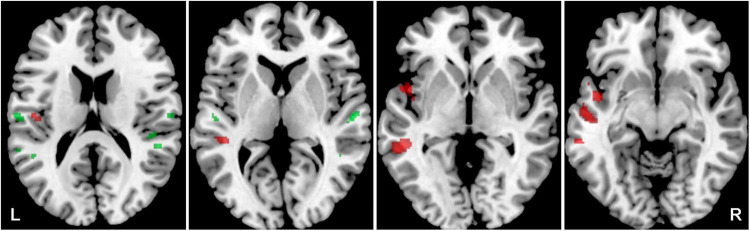
Cortical activation by a combination of all acoustic stimuli within the mask of AC, the comparison of Y-NH-T vs. Y-NH-NT. The comparison of tinnitus > no tinnitus in red (structures: left middle temporal gyrus), no tinnitus > tinnitus in green (structures: right middle temporal gyrus, transversal temporal gyrus bilaterally), *p* = 0.05 uncorrected.

The presence of tinnitus also led to changes in the activation of the left and right ACs. When tinnitus was present (Y-NH-T + O-NH-T + O-HL-T > Y-NH-NT + O-NH-NT + O-HL-NT) greater activation by word + babble was in the left AC (LI = 0.069), whereas the absence of tinnitus (Y-NH-NT + O-NH-NT + O-HL-NT > Y-NH-T + O-NH-T + O-HL-T) led to greater activation in the right AC (LI = –0.3). This is also true regardless of the of the acoustic stimulus (word: Y-NH-T > Y-NH-NT, LI = 0.32, Y-NH-NT > Y-NH-T, LI = –0.19; word + babble: Y-NH-T > Y-NH-NT, LI = 0.38, Y-NH-NT > Y-NH-T, LI = –0.069) although the extremity of the values changes (Figure 6).

### The effect of hearing loss

**FIGURE 7:**
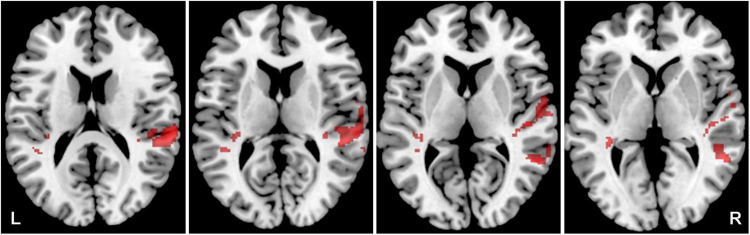
Cortical activation by a combination of all acoustic stimuli within the mask of AC, a comparison of the elderly with normal hearing (O-NH-NT + O-NH-T) vs. elderly with hearing loss (O-HL-NT + O-HL-T) groups. A comparison of normal hearing > hearing loss in red (structures: right planum temporale), hearing loss > normal hearing in green (structures: none), *p* = 0.05 uncorrected.

The aim was to identify the effect of hearing loss on the activity within both ACs (the LI was calculated) evoked by acoustic stimulation (all four types of stimuli). The effect of hearing loss was studied in the elderly population (O-NH-NT + O-NH-T > O-HL-NT + O-HL-T, *p* = 0.05, uncorrected) and showed increased activation (all 4 stimuli combined) within AC bilaterally (specifically primary AC, and in the right planum temporale) (Figure 7 and Table 3) in elderly patients with normal hearing compared to elderly patients with hearing loss (expressed presbycusis).

**TABLE 3:** The effect of hearing loss in elderly groups (presbycusis) on the evoked activity within the structures of AC mask.

	Cluster-level		Peak-level		Peak position		
	*P* uncorr	*K*_*E*_	*P* uncorr	*T*	*X*	*Y*	*Z*
**O-NH-NT + 0-NH-T** ** >** **O-HL-NT + O-HL-T**							
Planum temporale R	0.002	576	0.000	4.79	62	–28	16
Middle temporal gyrus R	0.043	220	0.000	3.75	54	–24	–14
Middle temporal gyrus R	0.128	116	0.000	4.39	52	–50	6
Transverse temporal gyrus L	0.180	88	0.005	2.67	–36	–34	4
**O-HL-NT + O-HL-T** ** >** **O-NH-NT + 0-NH-T**							

The comparison of elderly groups with normal hearing vs. elderly groups with hearing loss shows regions with increased activation in the elderly with normal hearing, whereas auditory stimulation in elderly patients with hearing loss did not produce any region with higher activity compared to normal hearing in the elderly. Differential statistical maps were masked with both described masks and uncorrected threshold of *p* = 0.05 with minimum cluster size of 30 voxels was used (*K_E_* is a cluster size expressed in voxels).

The effect of hearing loss (O-NH-NT + O-NH-T vs. O-HL-NT + O-HL-T) on the evoked activity (all four stimuli combined) in both ACs and their involvement in processing the auditory information led to decreased asymmetry toward the right AC (LI = –0.068) in the elderly with expressed presbycusis (O-HL-NT + O-HL-T), compared to LI = –0.17 in the elderly with normal hearing (O-NH-NT + O-NH-T).

### Concomitant visual and acoustic stimulation

**FIGURE 8:**
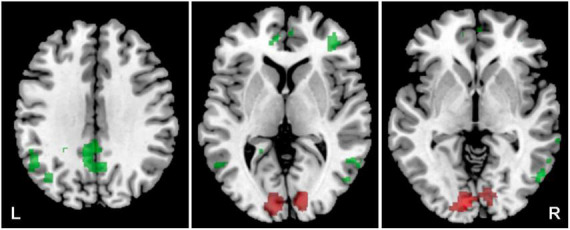
Cortical activation was evoked by a combination of the word + babble + visual stimulus. In the case of congruent visual stimulation, the activation is in green (structures of default mode network), incongruent in red (visual cortex), *p* = 0.001 incorrected.

The aim of using a combination of visual and acoustic stimulation was to identify how the supportive audio-visual information (congruent stimulation) alters the fMRI measured activity specifically within both ACs (the LI was calculated), compared to the confusing audio-visual information (incongruent stimulation), and whether the difference, if present, is based on the clarity of stimulus (word vs. word + babble). The combination of both acoustic (word, word + babble) and visual stimulation (congruent, incongruent) led to the activation of the regions outside the ACs, therefore for the analysis of activity generated by both types of stimulation, we did not use any predefined ROIs (whole brain data was used). The comparison of congruent vs. incongruent visual stimulation along the acoustic stimulus (word, word + babble), showed increased activation within the visual cortex for the combination of acoustic and incongruent visual stimuli (*p* = 0.05, FWE) and, to a lesser extent, also the right AC (*p* = 0.001, uncorrected). The combination of congruent visual and acoustic stimulation showed increased activation within the default mode network (DMN; specifically in the following structures: posterior cingulum, left angular gyrus, left and right middle temporal gyrus, small part of the anterior cingulum; *p* = 0.001, uncorrected), and also in the right middle frontal gyrus (*p* = 0.001, uncorrected) (Figure 8 and Table 4).

**TABLE 4:** The effect of congruent vs. incongruent audiovisual stimulation on the evoked activity within the cortex (whole brain approach).

	Cluster-level		Peak-level		Peak position		
	*P* FWE-corr	*K*_*E*_	*P* FWE-corr	*T*	*X*	*Y*	*Z*
**Incongruent** ** >** **Congruent**							
Calacarine cortex L, R	0.000	1231	0.000	7.68	–8	–90	–2
**Congruent** ** >** **Incongruent**							
Angular gyrus R	0.000	289	0.005	5.95	54	–68	–2
Angular gyrus L	0.000	213	0.138	5.08	–50	–66	18
Precuneus L, R	0.000	748	0.036	5.49	4	–56	12
Thalamus	0.000	263	0.048	5.40	–4	–2	10
Inferior frontal gyrus R	0.043	65	0.653	4.48	40	42	2

Data show where incongruent type of stimuli led to more significant activation than congruent type (upper part) and on the contrary where congruent activated more than incongruent type of stimulus. Differential statistical maps were used with uncorrected threshold of *p* = 0.001 and minimum cluster size of 50 voxels (*K_E_* is a cluster size expressed in voxels).

The presence of supportive visual stimulus (congruent) along the acoustic stimulation (word) led to a greater involvement of the right AC (LI = –0.19), compared to the combination with incongruent visual stimulus (LI = –0.082). Similar findings were present in the case of the unclear acoustic stimulus (word + babble), with congruent (LI = –0.12) and incongruent (LI = –0.033) visual stimulation, but in both cases of unclear acoustic stimulation the LI was closer to 0 suggesting more even involvement of both ACs.

## Discussion

The aim of this study was to investigate the effect of tinnitus, aging and hearing loss in the elderly (presbycusis) on the function of the auditory cortex and related structures during complex acoustic stimulation. In addition to the AC, we also focused on the Amg, HP, and Ins, subcortical structures that were identified as a part of the tinnitus network in our previous morphometric study (Profant et al., 2020). The auditory testing and anamnestic data clearly defined the groups based on their age, presence of tinnitus and hearing loss. Auditory results showed significant differences between the groups with and without hearing loss as well as between young and old groups in pure tone audiometry, and also in speech tests. Tinnitus had no effect on hearing abilities in the examined auditory functions. The main findings of our study, in relation to the complexity of the stimulus demonstrated, were that for more complex stimulus there is greater asymmetry in the ACs activation toward the left cortex, whereas clearer stimulus leads to rightward asymmetry of the ACs activation. The effect of aging was most apparent by the greater left AC involvement in the processing of acoustic signals, however, overall the aging led to more even activation of both ACs (LI closer to 0). The effect of hearing loss in the aged population (degree of presbycusis) showed a decreased amount of activated AC, with more even involvement of both ACs in the auditory processing (LI closer to 0) in the population with expressed presbycusis. The combination of acoustic and visual stimulation led to a greater activation of the visual cortex when both stimuli were incongruent, compared to the congruent situation. On the other hand, the congruent stimulation activated structures of the default mode network. Overall, the congruent stimulation increased the involvement of the right AC, whereas the incongruent stimulation activated both ACs more evenly (LI closer to 0).

The function of the AC in the elderly is influenced by several factors. The hypofunctional periphery (a clear cause of presbycusis) leads to a deteriorated coding of the acoustic signal, and therefore to an altered stimulation of the central structures (Schuknecht and Gacek, 1993). Regardless of the cochlear pathology, aging is also accompanied by the altered function of the subcortical pathway at different levels, resulting in an alteration of the sound evoked neuronal signal (Profant et al., 2019) along with a white matter structure within the pathway (Profant et al., 2014). Finally, the function and structure of ACs is influenced by aging (Profant et al., 2015; Glick and Sharma, 2017; Rosemann et al., 2020). Another factor that affects the processing of complex sounds is cognition, which is altered by aging as well as by hearing loss. Tinnitus adds another dimension to the alteration of the auditory function. As described in our previous paper by Bureš et al. (2019), tinnitus leads not only to deteriorated hearing ability, but to some extent, also improves some auditory function. Although there are no reports that tinnitus would disappear over time, the deterioration of tinnitus intensity, as well as the change from unilateral to bilateral tinnitus, was reported by Refat et al. (2021). An interesting fact in this study was also the increased annoyance by regular sound over time in tinnitus patients, which offers a potential explanation of our finding of changes in the BOLD activation within the AC. The combination of tinnitus and presbycusis, the most common combination of hearing pathologies in the elderly, provides an intriguing opportunity to identify changes within the AC and structures of the tinnitus network.

### The auditory function

The analysis of our audiometric results showed a clear difference caused by aging, and constituted a basis for the selection criteria. Increasing age led to a significant difference between both the young groups and all the elderly groups in the auditory thresholds, SRT and SIN, but also significant differences in the auditory thresholds, SRT and SIN, were present between the elderly groups with normal hearing and those with expressed presbycusis. Presbycusis not only leads to inadequate coding within the cochleae (as represented by auditory threshold), but also at different levels of the auditory pathway. The main deficiency appears to be in the coding of temporal parameters of sound (Profant et al., 2019), one of the most important factors in speech processing (Fogerty et al., 2015; Moore, 2016). This pronounced idea is supported by the positive correlation between the deteriorated temporal processing and altered speech processing in the elderly (Pichora-Fuller and Singh, 2006; Profant et al., 2019).

Tinnitus did not alter hearing abilities when the adequate tinnitus and non-tinnitus groups were compared, this was also the case for speech in noise testing. The elderly participants showed only a minimal effect of aging on cognition, as shown by the averages of MoCA results, enabling us to exclude the cognitive influence from the observed changes. However, MoCA is only a screening test and, as previously reviewed by Fuksa et al. (2021), standard cognitive tests are not an optimal tool for identifying the impact of mild cognitive impairment on hearing ability (Wayne and Johnsrude, 2015). The level of annoyance of tinnitus was equally low among the groups, therefore we can only assume a minimal impact of emotional distress caused by tinnitus on our data. Refat et al. (2021) reported a very close relationship between tinnitus distress and hyperacusis, however, none of our participants reported hyperacusis, which might be one of the explanations why, overall, tinnitus annoyance was low.

### The effect of complexity of the acoustic stimulus and aging on the auditory cortices activation

Auditory stimulation evoked measurable BOLD activity in bilateral ACs by all four different stimuli. AC is the primary recipient of neuronal signals induced by acoustic stimulation; however, the acoustic signal is coded into the neuronal signal in the cochlea, and then shaped at different levels of the auditory pathway before it reaches the AC.

Our data showed that the complex (either speech or speech like sounds) acoustic stimuli activated the right AC slightly more than the left, and the asymmetry did not change with aging, although it became less extreme (LI closer to 0). In our previous study (Profant et al., 2015), tonal stimulation led to an increase of activation in the right AC with aging; respectively the amount of activation became similarly equal to our current data of activity evoked by complex stimuli.

An interesting outcome was presented when the activation by different stimuli was analyzed separately (data from all groups were pooled together). There was a clear difference in the involvement of the left and right AC that depended on the stimulus; the most complex stimulus (babble + word) increased the involvement of the left AC, whereas the simpler stimulus (word) increased the involvement of the right AC. This finding might initially appear to be in disagreement with the accepted dominance of the left hemisphere in speech processing (McGettigan and Scott, 2012), but word + bubble is the most challenging for temporal processing, and, therefore, the left dominance agrees with the theory of left AC domination in the temporal processing. The activation with a babble also shifts the LI toward the left hemisphere but, to a lesser extent, than the word + babble. Non-word and word both lead to a right hemisphere dominance. In the case of word it is particularly surprising, however, this could potentially be explained by aging and the greater involvement of the right AC, as shown by our previous project when stimulation by tones was involved (Profant et al., 2015). The potential effect of aging on the increased involvement of the right AC is also supported by our data from young healthy participants when intelligible stimulus (word) overall activates more left AC, suggesting that age alters the involvement of both hemispheres. A further explanation of the effect of different acoustic stimulation on the left/right AC involvement in the auditory processing could be that a more auditory challenging stimulation (babble + word), or rather phonetically meaningless stimulation (babble, non-word) that are cognitively more challenging, leads to an increased effort to understand the meaning (volunteers did not know what kind of stimulus would be presented) and therefore an increased involvement of the left AC. Zatorre and Belin (2001) showed that the left hemisphere does in fact react to fast and slow transition, and the right superior temporal gyrus only to slow transition; this is supported by the data by Boemio et al. (2005) that the left side analyzes signals on a time scale of 25–50 ms, whereas the right side on a time scale of 200–300 ms. Specht et al. (2009) showed that the activity of the right AC was evoked by all acoustic stimuli (seven step manipulation of white noise perturbation in sound that gradually changed toward either speech or music sounds) irrespective of their acoustic properties (sensitive to manipulation, but not specific to the type of sound stimulation). This finding suggests that the right temporal lobe is more sensitive to acoustic manipulation within sounds. This could be a potential explanation of the greater right AC involvement in the elderly that loses the ability to track fast temporal changes, which could be compensated by utilization of the slower acoustic manipulation. Increased right AC activation caused by aging, without a decrease in the left AC activation, was found in our previous study that used only narrow band stimuli centered around tonal frequencies in the elderly (no effect of hearing loss was present) (Profant et al., 2015). Keller et al. (2019) observed in a similar way, that the hemispheric specialization becomes less specific with aging, especially in the case of slowly changing speech cues. This observation is also supported by findings in EEG recorded activity that is higher in the right temporal lobe of the elderly (Giroud et al., 2017), suggesting increased employment of the right AC in the elderly population and potentially increased importance of slowly changing acoustic cues important for prosodic and intonation patterns.

The changes related to aging are not only bound to function, but also to structure with a decrease in the cortical thickness and volume that are also present in the AC, however, they cannot be linked to age related hearing loss (Profant et al., 2014; Eckert et al., 2019). A similar pattern (the effect of aging but not of hearing loss) is also present in the levels of chemical substances in the AC (Profant et al., 2013).

### The effect of presbycusis on the auditory cortices processing

The effect of hearing loss in the elderly population (presbycusis) led to decreased activation within the AC in the elderly population with hearing loss compared to the elderly population with normal hearing bilaterally, and also to decreased asymmetry of AC activation (LI closer to 0) in the elderly groups with hearing loss regardless of acoustic stimuli. The decreased activation of the AC (specifically in the right planum temporale) due to hearing loss, contrasts with our previous findings (Profant et al., 2015) where the degree of presbycusis did not produce any difference in the evoked activity within the AC. One explanation could be the complex acoustic stimulation used in the current study compared to narrow band noise centered around sound frequency used in Profant et al. (2015). The main difference in the auditory thresholds between the elderly groups is in high frequencies, which were rarely present in our complex stimuli. In addition, expressed presbycusis is more pronounced in the processing of complex rather than tonal stimuli. As aforementioned, presbycusis negatively affects temporal coding, an important auditory feature linked to the processing of speech, that partially takes place within the AC, as proved by the gap detection test and lesions of the AC (Syka, 2002). Previous experiments even showed that the temporal processing is tied to the left AC (Zaehle et al., 2004), whereas the right AC processes spectral information (Tramo et al., 2002, 2005; Schönwiesner et al., 2007). Although it is believed that speech processing is linked to the left AC, a profound analysis by McGettigan and Scott (2012) showed that it is not as straightforward and, in the case of unintelligible stimuli (even though amplitude and spectral modulation was identical with intelligible speech), led to higher right AC activation (Rosen et al., 2011).

### The presence of tinnitus

The presence of tinnitus led to an increase as well as a decrease of activity during acoustic stimulation (all four stimuli) in specific parts of the AC bilaterally. However, when the involvement of the left vs. right AC was compared between the groups with and without tinnitus, the activation by acoustic stimuli showed that the tinnitus presence shifted the activity more toward the left AC, whereas tinnitus absence more toward the right AC. The presence of tinnitus also led to increased activity during acoustic stimulation in the non-auditory structures (Ins and HP).

The previously mentioned theory regarding the clarity of stimulus and cognition, may be supported by our observation in volunteers with tinnitus. The tinnitus effect on cognition is based on the load theory (Lavie et al., 2004). Tinnitus acts as a distractor, that decreases the overall processing capacity especially during the multitasking paradigms and overload of cognitive resources (Khan and Husain, 2020). Tinnitus, therefore, leads to an increased effort needed to maintain attention (Hallam et al., 2004), and the suboptimal performance of the working memory (Cardon et al., 2019). The optimal function of the working memory is an important factor in speech perception, especially under strenuous conditions (Nuesse et al., 2019), therefore tinnitus could potentially worsen such a situation.

Tinnitus affects hearing ability to some degree, negatively influences the ability to identify tones in noise, and positively affects sensitivity to interaural time differences (Bureš et al., 2019). Although some authors advocate for the negative effect of tinnitus on speech processing abilities (Moon et al., 2015; Gilles et al., 2016), our previous work (Bureš et al., 2019) could not confirm the direct link between the presence of tinnitus and worsened speech perception although in patients with tinnitus, speech perception is more dependent on the sensitivity to temporal modulation and interaural time delay. During the speech processing under difficult conditions, the dependency on temporal parameters becomes even more essential as described by Bureš et al. (2019) and suggests a higher involvement of the left AC. Our observations support this idea since the presence of tinnitus leads to an increased involvement of structures in the left temporal lobe, and the comparison of LIs showed a shifted activation toward the left AC in the presence of tinnitus, and toward the right AC in its absence (except for healthy young individuals). Berlot et al. (2020) found only minimal changes in the auditory evoked BOLD activation of the AC, which is not necessarily a contradictory finding to ours, because from our data we cannot conclude that the overall amount of evoked activity increased in tinnitus patients (this is true only for some studied regions and vice versa in the non-tinnitus groups), only that the activity was shifted more toward the left AC in tinnitus patients and more toward the right AC in non-tinnitus patients. Another difference was represented by the acoustic stimuli that were less complex in the study by Berlot et al. (2020), and also that the volunteers included in the study suffered from unilateral tinnitus. Our data also propose a hypothesis that tinnitus functions as a distractor that shifts the LI toward the greater involvement of the left AC, similarly to the effect of stimulus clarity. Since tinnitus laterality is evenly distributed among our volunteers, the greater affinity of the left temporal lobe structures to tinnitus is not a result of the tinnitus laterality. Reduced and delayed wave V on ABR in tinnitus patients is another finding that supports the idea that tinnitus reduces contrast-amplification circuits that amplify relevant, and ignore irrelevant, stimuli (Knipper et al., 2020). On the other hand, Hofmeier et al. (2018), reported a decreased BOLD fMRI response in auditory structures caused by auditory stimulation in volunteers with tinnitus. The different results compared to our findings, might be due to the less complex auditory stimuli and age of participants in the study by Hofmeier et al. (2018). An increased metabolic activity in the left AC of tinnitus patients was also reported by Schecklmann et al. (2011) and to some degree also by Geven et al. (2014).

In our previous experiments (Profant et al., 2020) we have identified subcortical structures where morphometric changes of the gray matter were related to the presence of tinnitus, specifically tinnitus led to an increased volume of the HP and Amg, and a borderline decrease in Ins. Insular involvement is linked to tinnitus distress as proposed by De Ridder et al. (2014). Chen et al. (2017) proposed a bidirectional connection between limbic structures and the temporal lobe. The increased activity found in Ins is in agreement with the findings of Hofmeier et al. (2018), however, their finding is more complex, suggesting that BOLD activity is increased in anterior Ins (due to tinnitus distress), and decreased in posterior Ins that is responsible for sound detection (Sadaghiani et al., 2009). Hofmeier et al. (2018) reported decreased activity in the HP, in opposition to our results. This difference can be explained by the different age of participants in both studies, resulting in more profound hearing loss and more complex auditory stimuli in our study, since HP is important for accentuating behaviorally important sounds (Kraus and White-Schwoch, 2015).

### Concomitant visual and acoustic stimulation

Congruent concomitant audiovisual stimulation resulted in increased activity in the structures that form the DMN, whereas incongruent stimulation led to an increased activation in the visual cortex. The overall activity within the bilateral ACs was not influenced when congruent and incongruent visual stimulus was present, however, the involvement of the left vs. right AC changed; congruent (clearer) stimulation shifted the activity more toward the right AC, whereas incongruent (unclear) stimulation caused equal involvement of both ACs which became even more clear when the word was replaced by word + babble as acoustic stimulus.

As previously mentioned, tinnitus works as a distractor (Khan and Husain, 2020). A similar case could be made with incongruent visual stimulation. On the other hand, congruent visual stimulation could potentially suppress the negative effect, since coordinated multisensory information enhances perceptual clarity and reduces ambiguity (Bischoff et al., 2007); it can also speed up reaction time (Forster et al., 2002). Musacchia and Schroeder (2009) identified neurons within the AC that respond to multisensory stimulation, and Bizley and King (2009) identified fields within the animal AC that can be influenced by visual stimulus. The greater activation within the DMN evoked by congruent stimuli supports the previously mentioned facts, suggesting a reduced need for attention due to the higher clarity of stimulus. Alternatively, greater visual cortex activity evoked by incongruent stimulation supports the idea by Wu et al. (2009) that visual stimulation accompanied by irrelevant auditory signal, results in enhanced visual perception that leads to increased activation of the visual cortex.

Although, tinnitus was previously confirmed to disrupt activity within the structures of a default mode network (Lanting et al., 2016), our data were not able to confirm this finding. Yet the effect of congruent vs. incongruent audiovisual stimulation when the visual stimulus acts as a supporter or distractor, suggest that supportive stimulation, which is easier to process, increases activity within the DMN structures. This finding indirectly confirms the negative effect of tinnitus (distractor) on activation and connectivity within the DMN.

## Conclusion

Our study revealed changes in the function of AC related to aging, degree of hearing loss (presbycusis), and the presence of tinnitus. Tinnitus presence led to a greater involvement of the left AC in the processing of complex sounds and a similar finding was also related to complexity of the stimulus, suggesting that more unclear stimulus (either caused by the presence of tinnitus or competing sound) increases the activation of the left AC. Tinnitus also caused increased activation within the structures of the limbic system that were previously identified as part of the tinnitus network. Aging led to increased involvement of the right AC in the auditory processing, and hearing loss led to decreased activation within the AC that was more evenly distributed bilaterally. Concomitant visual and acoustic stimulation caused changes of the activation dependent on congruency (default mode network) or discongruency (visual cortex) of visual stimulation. The complexity of acoustic stimulus was another factor that influenced the asymmetry of the left/right AC activation, however, this was also partly dependent on the age of the participants.

Overall, we can conclude that the complex acoustic stimulation is better suited for identification of hearing loss related central changes than simple tonal stimulation, and that the complexity of the stimulus, presbycusis and tinnitus alters the left/right involvement of the AC. Our reported findings support the idea of age-related changes within the function of central auditory structures that can be only partially explained by peripheral hearing loss that are signs of the central presbycusis.

## Data availability statement

The raw data supporting the conclusions of this article will be made available by the authors, without undue reservation.

## Ethics statement

The studies involving human participants were reviewed and approved by Ethics Committee of the University Hospital Motol, in Prague. The patients/participants provided their written informed consent to participate in this study.

## Author contributions

JF and OP: manuscript preparation, examination, auditory examination, statistical analysis, and work load cca 20%. JT: MR examination, statistical analysis, manuscript preparation, and work load cca 20%. VS: auditory examination, database administration, and work load cca 13%. DT: auditory examination, database administration, and work load cca 12%. AŠ: MR examination, statistical analysis, and work load cca 5%. JS: manuscript preparation, project overview, and work load 10%. All authors contributed to the article and approved the submitted version.

## Abbreviations

AC: auditory cortex
Amg: amygdala
O-HL-NT: aged group with expressed presbycusis
HP: hippocampus
Ins: anterior insula
LI: lateralization index
DMN: default mode network
O-NH-NT: aged group with mild presbycusis
PTAV: pure tone average
SNHL: sensorineural hearing loss
O-HL-T: aged group with tinnitus and expressed presbycusis
THI: Tinnitus Handicap Inventory questionnaire
O-NH-T: aged group with tinnitus and mild presbycusis
Y-NH-T: young group with normal hearing and tinnitus
Y-NH-NT: young control group.
